# An Unusual Presentation of Giant Cell Arteritis

**DOI:** 10.1155/2012/498174

**Published:** 2012-07-03

**Authors:** Siddhesh Prabhavalkar, Pawel Bogusz, Reena Merard, Mark Gormley

**Affiliations:** ^1^Mater Infirmorum Hospital, Belfast Health and Social Care Trust, Belfast BT9 7AB, UK; ^2^Department of Histopathology, Royal Victoria Hospital, Belfast BT12 6BA, UK

## Abstract

Giant cell arteritis (GCA) is a chronic vasculitis that typically presents with headache, fever and polymyalgia although atypical presentations are known. We present a case of GCA with nonproductive cough and pyrexia of unknown origin emphasizing this atypical nature of presentation. We report a rare association of GCA with granulomatous hepatitis. We also support the use of PET scanning in diagnosing and monitoring this condition.

## 1. Introduction

Giant cell arteritis (GCA) is a systemic granulomatous vasculitis affecting large and medium-sized arteries [1]. Typical clinical features may include fever, headache, jaw claudication, palpable and tender temporal arteries, symptoms of polymyalgia rheumatica (PMR), visual disturbance, and raised serologic inflammatory markers such as the erythrocyte sedimentation rate (ESR) or C-reactive protein (CRP) [2]. In the absence of typical signs and symptoms, diagnosis becomes difficult, thus increasing the risk of severe complications like visual loss. We report an unusual presentation of GCA with nonproductive cough, pyrexia of unknown origin (PUO), and in the absence of typical symptoms of GCA or a diagnosis of PMR.

## 2. Case Report

A 60-year-old female presented with 4-week history initially of dry cough and night sweats with subsequent nausea and weight loss. Her past medical history, social and occupational history, and systematic questioning did not reveal further relevant information; neither headache nor visual disturbance was noted. Her drug history included Rosuvastatin 10 mg daily, Glucosamine 500 mg thrice daily, Ibuprofen, and Salbutamol inhaler as required.

She had a temperature of 39°C and physical examination was unremarkable. There was no temporal tenderness and no limb girdle tenderness or weakness. Fundoscopy showed normal vessels.

Initial laboratory tests showed raised CRP 335 mg/L and ESR 62 mm/hr with elevated liver enzymes showing a mixed picture with total Bilirubin 8 *μ*mol/L, ALP 589 U/L, GGT514 U/L, ALT 112 U/L, AST 47 U/L, and albumin 36 g/L. Chest X-ray was reported as normal.

Ultrasound scan (USS) of abdomen showed a stone in the gall bladder with a normal common bile duct. Magnetic resonance cholangiopancreatography (MRCP) confirmed USS findings and showed a normal biliary tree. Her atypical infective screen was negative including blood cultures, and a serologic screen for viruses, atypical bacteria, and fungi. Her liver screen including hepatitis serology and autoantibody screen was negative.

CT scan of chest, abdomen, and pelvis showed a spiculated nodule in the posterior region of the apex in the left upper lobe of the lung. Bronchoscopy was unremarkable and the washings of left upper lobe lesion were negative. The lesion was considered to be longstanding and related to previous resolved inflammation.

**Figure 1:**
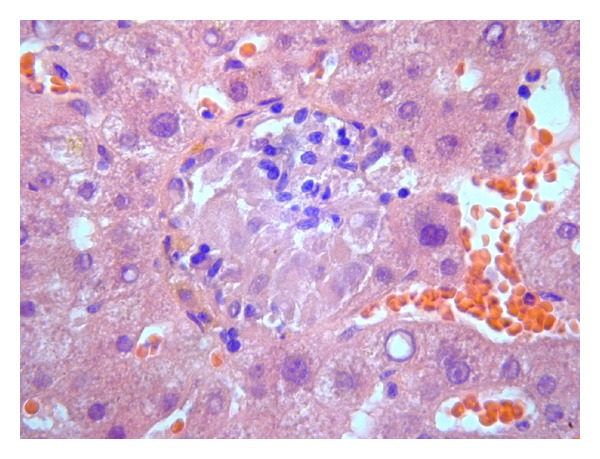
Liver biopsy showing hepatic granuloma.

She continued to be pyrexic with worsening liver functions. She then underwent liver biopsy which showed lobular granulomatous inflammation with no significant Kupfer activity or inclusions (Figure 1).

**Figure 2:**
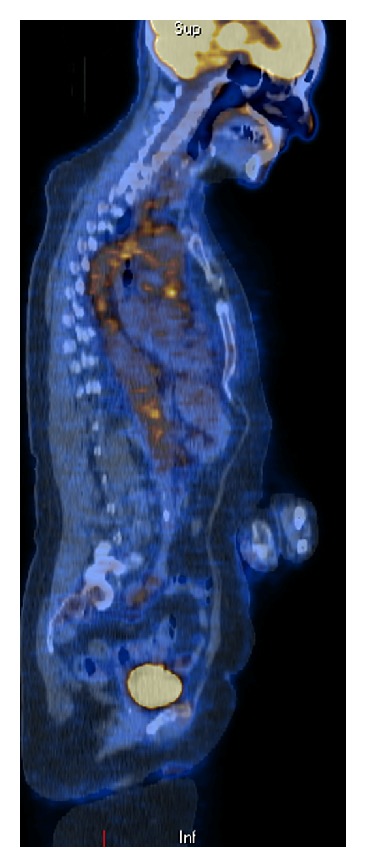
PET scan image showing hot spots in the central large vessels.

**Figure 3:**
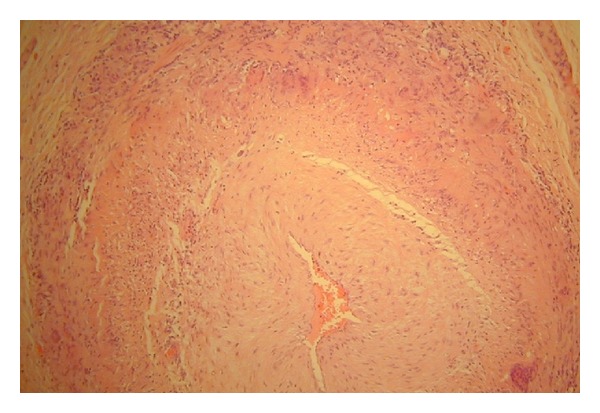
Temporal artery biopsy showing arteritis.

A whole body positron emission tomogram (PET) showed increased metabolic activity within central large vessels; thoracic and abdominal aorta; proximal carotids and subclavian arteries (Figure 2). These findings were strongly suspicious of a large vessel vasculitis. Temporal artery biopsy confirmed giant cell arteritis (Figure 3).

Treatment with steroids resulted in prompt resolution of her symptoms and normalizing of CRP, ESR, and liver functions. A followup PET scan in 2 months showed complete resolution of previous abnormalities confirming a positive response to treatment.

## 3. Discussion

In the absence of characteristic signs and symptoms, diagnosing GCA can pose a challenge. Approximately 10 percent of patients with GCA have upper respiratory tract symptoms. These include a cough that is nonproductive and leads to significant diagnostic confusion [3]. Cough associated with GCA is thought to result from vasculitis in the area of cough receptors, which are located throughout the respiratory tree. Fever occurs in up to one-half of patients with GCA and is usually low grade. However, in approximately 15 percent of patients, the fevers exceed 39°C; often leading to misdiagnoses of infections [4]. Elevated serum concentrations of hepatic enzymes, such as aspartate aminotransferase and alkaline phosphatase, occur in 25 to 35 percent of patients [5]. The elevations are typically modest and revert to normal with glucocorticoid therapy. Previous studies of liver biopsy have shown nonspecific changes.

The present case reinforces the importance of suspecting GCA as cause of PUO especially in the absence of typical symptoms like headache and vision loss. It also demonstrates an association between GCA and granulomatous hepatitis. This is the first reported case in Northern Ireland demonstrating this association [6]. As confirmed in a recent meta-analysis [7], we also support the use of PET scanning as a diagnostic tool for suspected GCA, especially in the absence of typical symptoms. PET scanning can also be used in assessing response to treatment.
